# ATG5-mediated inducible autophagy sustains CAR-T cell durability under solid tumor stress

**DOI:** 10.3389/fimmu.2026.1720544

**Published:** 2026-04-22

**Authors:** Sang-Eun Jung, Minji Lim, Hyungwoo Jeong, Youngchae Moon, Hyungseok Seo

**Affiliations:** 1 Research Institute of Pharmaceutical Sciences, Seoul National University, Seoul, Republic of Korea; 2 Laboratory of Cell & Gene Therapy, College of Pharmacy, Seoul National University, Seoul, Republic of Korea

**Keywords:** ATG5 overexpression, autophagy, CAR-T cell therapy, CAR-T persistence, tumor microenvironment

## Abstract

Autophagy functions as a context-dependent stress adaptation pathway in T cells; however, its role in sustaining chimeric antigen receptor (CAR)-T cell function within solid tumor environments remains insufficiently defined. In this study, we investigated whether ATG5-mediated autophagy regulation contributes to CAR-T cell functional durability under tumor-associated stress conditions. ATG5 overexpression (OE) CAR-T cells did not increase basal autophagy activity but instead selectively enhanced autophagy flux in response to inducible stimuli. Under tumor-mimicking immunosuppressive conditions, ATG5 OE CAR-T cells maintained cytotoxic activity during prolonged antigen exposure and exhibited preserved effector cytokine production together with reduced oxidative stress. Consistent with these *in vitro* findings, ATG5 OE CAR-T cells exhibited enhanced antitumor efficacy *in vivo* under IR-preconditioned settings, characterized by improved tumor control and survival, which was associated with sustained effector function of tumor-infiltrating CAR-T cells. Collectively, these findings demonstrate that reinforcing inducible autophagy capacity through ATG5 promotes the maintenance of CAR-T cell function under tumor-associated challenges, highlighting a targeted strategy to enhance CAR-T cell persistence in solid tumor immunotherapy.

## Introduction

Chimeric antigen receptor (CAR)-T cell therapy has achieved transformative outcomes in hematological malignancies; however, its efficacy in solid tumors remains substantially limited. This discrepancy is primarily attributed to the immunosuppressive tumor microenvironment (TME), which restricts CAR-T cell trafficking, promotes functional exhaustion, and compromises persistence, thereby diminishing sustained therapeutic benefit (1–3). Within the TME, hypoxia, nutrient deprivation, and excessive accumulation of reactive oxygen species (ROS) impose profound metabolic constraints that erode effector T cell cytotoxicity and survival (4, 5).

To overcome these barriers, increasing attention has shifted toward intrinsic regulatory programs that enable T cells to maintain functional stability under hostile conditions. Among such mechanisms, autophagy has emerged as a central homeostatic process. By facilitating the selective removal of damaged organelles and maintaining cellular energy balance, autophagy supports T cell survival, preserves mitochondrial integrity, and sustains overall cellular fitness (6–8).

Beyond its canonical degradative role, autophagy has been increasingly recognized as a modulator of T cell functional states. By maintaining mitochondrial metabolic capacity and limiting stress-induced dysfunction, autophagy contributes to the preservation of effector competence under prolonged stimulation (9). Importantly, its functional impact is highly context dependent, shaped by environmental cues and activation states (10–12). Autophagic activity is dynamically regulated by cytokine signaling and metabolic stress, being suppressed in highly inflammatory conditions while relatively preserved under conditions that favor prolonged functional maintenance (13). In this regard, autophagy has been proposed as a metabolic checkpoint that regulates intracellular nutrient availability and mitochondrial quality control—processes that are particularly critical in nutrient-restricted environments such as the TME (14).

Consistent with this framework, genetic ablation of essential autophagy regulators such as ATG5 or ATG7 impairs T cell fitness and accelerates dysfunction in tumor-associated contexts (15). Conversely, enforced autophagy activation has been reported to attenuate exhaustion-associated phenotypes and support sustained effector function in tumor-infiltrating lymphocytes (TILs) (16, 17). Notably, ATG5 overexpression (OE) has been reported to enhance metabolic resilience and functional durability in conventional CD8^+^ T cells (8, 18). However, these observations have largely been derived from non-engineered T cell systems, hematological malignancies, or simplified *in vitro* stress models. Whether autophagy modulation confers similar functional advantages in engineered CAR-T cells—particularly within the complex and restrictive microenvironment of solid tumors—remains poorly defined.

Addressing this gap is especially timely, as current strategies to improve CAR-T cell efficacy in solid tumors have focused predominantly on enhancing tumor trafficking or modifying antigen recognition, while comparatively less attention has been paid to intrinsic mechanisms that sustain CAR-T cell function after tumor entry. In this context, whether reinforcement of autophagy can stabilize CAR-T cell effector competence under solid tumor–associated stress—independent of effects on infiltration or terminal differentiation—remains a critical unresolved question.

To directly address this issue, we investigated whether ATG5-mediated autophagy modulation supports CAR-T cell functional persistence under solid tumor–associated stress. We generated ATG5 OE CAR-T cells and evaluated their functional properties in a murine solid tumor model under irradiated (IR) and non-irradiated (Non-IR) conditions, which differ in tumor accessibility. By integrating analyses of inducible autophagy flux, effector durability, exhaustion-associated phenotypes, and antitumor efficacy, this study delineates a context-dependent role of ATG5-driven autophagy regulation in sustaining CAR-T cell function within the solid TME.

## Materials and methods

### Mice

C57BL/6J mice (5 weeks old, originally from Jackson Laboratory, stock #000664) were purchased from JA BIO Inc. (Suwon, Republic of Korea). All mice were rested for at least one week before being used in experiments, and 6-week-old mice were used for all procedures. Age-matched cohorts were maintained under specific pathogen-free conditions in the animal facility at Seoul National University, with controlled temperature (23 ± 1 °C), humidity (55 ± 10%), and a 12-hour light/dark cycle. All procedures were conducted under IACUC-approved protocols (SNU-240528-4). Animals were provided ad libitum access to sterile chow and water.

### Mouse CD8^+^ T cell isolation and activation

Spleens were collected aseptically from 6-week-old male C57BL/6J mice (Jackson Laboratory, stock #000664) maintained under specific pathogen-free conditions at Seoul National University. Immediately following dissection, spleens were placed in ice-cold MACS buffer consisting of phosphate-buffered saline (PBS) supplemented with 2% fetal bovine serum (FBS; Gibco, Waltham, MA, USA) and 2.5 mM ethylenediaminetetraacetic acid (EDTA). To generate single-cell suspensions, spleens were mechanically dissociated using a 70 μm cell strainer (SPL Life Sciences, Pocheon, Republic of Korea) and the plunger of a sterile syringe under aseptic conditions. Red blood cells were removed by treating the cell suspension with ACK lysing buffer (A10492-01, Gibco) for 2 minutes on ice, followed by immediate neutralization and washing with excess cold MACS buffer to preserve lymphocyte viability. After centrifugation (400 × g, 5 min, 4 °C), the leukocyte pellet was resuspended in MACS buffer and subjected to magnetic negative selection for CD8^+^ T cells using the EasySep™ Mouse CD8^+^ T Cell Isolation Kit (19853, STEMCELL Technologies, Vancouver, BC, Canada) according to the manufacturer’s instructions. Briefly, the leukocyte pellet was resuspended in ice-cold MACS buffer and the suspension was transferred into a 14 mL polystyrene tube. Fc receptor blocking reagent (FcR blocker) was added at a concentration of 20 μL/ mL of sample and gently mixed. Subsequently, 50 μL/mL of the Mouse CD8^+^ T Cell Isolation Cocktail (19853, EasySep™, STEMCELL Technologies) was added, and the sample was incubated at room temperature (RT) for 10 minutes. After incubation, RapidSpheres™ magnetic particles were added to the sample at 125 μL/mL. The mixture was gently mixed and incubated at RT for an additional 5 minutes. The total volume was then adjusted by adding MACS buffer to a final volume of 5 mL. The tube was placed into the EasyEights™ magnet (18103, STEMCELL Technologies) without the cap and incubated at RT for 5 minutes to allow magnetically labeled non-CD8^+^ cells to be retained along the wall of the tube. The enriched CD8^+^ T cells, remaining in suspension, were carefully collected by pipetting into a new sterile tube. The isolated cells were immediately counted and assessed for viability using trypan blue exclusion and subsequently used for downstream activation and transduction. Cells were resuspended in RPMI-1640 medium supplemented with 10% FBS, 1% penicillin-streptomycin (Gibco), 2 mM L-glutamine, and 50 μM 2-mercaptoethanol, and plated in 24-well plates pre-coated with monoclonal anti-mouse CD3ϵ (5 μg/mL; BE0001-1, Bio X Cell, Lebanon, NH, USA) and anti-mouse CD28 (5 μg/mL; BE0015-1, Bio X Cell) antibodies. Activation was carried out for 24 hours at 37 °C in a humidified incubator with 5% CO_2_ prior to retroviral transduction with CAR constructs.

### Generation of retroviral constructs

To investigate the effects of autophagy modulation in CAR-T cells, retroviral vectors encoding murine Atg5 or LC3b were constructed. ATG5 was selected as a key upstream regulator of autophagosome formation, whereas LC3b represents a downstream structural component of the autophagosome, allowing comparative assessment of autophagy regulation at distinct functional levels (19). Synthetic cDNA sequences encoding full-length murine ATG5 (Uniprot ID: Q99J83) and LC3b (Uniprot ID: Q9CQV6) were synthesized as gBlocks^®^ Gene Fragments (Integrated DNA Technologies, Coralville, IA, USA). The gBlocks were inserted into the pMSCV-IRES-enhanced green fluorescent protein (eGFP) retroviral backbone (Addgene plasmid #27490), which allows for bicistronic expression of the gene of interest and eGFP as a reporter marker. Assembly was performed using the NEBuilder^®^ HiFi DNA Assembly Kit (E2621L, New England Biolabs, Ipswich, MA, USA), following the manufacturer’s instructions. Correct insertion and sequence fidelity were confirmed by Sanger sequencing (Macrogen, Seoul, Republic of Korea). The amino acid sequences of ATG5 and LC3b used in this study are listed in **Supplementary Table 1**.

For CAR construct generation, a second retroviral vector targeting human CD19 (hCD19) was assembled. This construct included a single-chain variable fragment (scFv) derived from the FMC63 monoclonal antibody, which specifically binds to hCD19. The scFv was linked to the transmembrane and intracellular signaling domains of murine CD28 and CD3ζ to enable T cell activation upon antigen engagement. To facilitate surface detection and monitoring of CAR expression, a Thy1.1 epitope tag was fused to the CAR construct via a P2A self-cleaving peptide sequence, enabling co-expression of Thy1.1 and the CAR protein from a single transcript. The full-length CAR construct was cloned into a MSCV-based retroviral backbone compatible with packaging in Platinum-E (Plat-E) cells. All plasmids were verified by sequencing prior to use in virus production.

### Cell lines and *in vitro* culture

The B16F0 (mouse melanoma) cell line was purchased from the American Type Culture Collection (ATCC), and the Plat-E (ecotropic retroviral packaging) cell line was obtained from Cell Biolabs (San Diego, CA, USA). B16F0-hCD19 cells were generated via transduction with an amphotropic virus encoding hCD19 followed by sorting for high expressers. All cell lines were cultured in DMEM (11995-073, Gibco) supplemented with 10% FBS and 1% penicillin-streptomycin at 37 °C in a humidified 5% CO_2_ incubator. Cells were confirmed to be mycoplasma-free and were used between passages 4 and 10 after thawing.

### Retrovirus production and transfection

Retroviral particles were generated using Plat-E packaging cells, which stably express the ecotropic envelope and gag/pol genes required for retrovirus production. Plat-E cells (3 × 10^6^) were seeded into 10 cm tissue culture-treated dishes (SPL Life Sciences) and cultured overnight in complete DMEM supplemented with 10% fetal bovine serum. On the following day, cells at ~70–80% confluence were transfected with retroviral vectors using the TransIT^®^-LT1 Transfection Reagent (MIR2306, Takara Bio USA, San Jose, CA, USA). For each 10 cm dish, a total of 15 μg of retroviral expression plasmid was mixed with 3.4 μg of pCL-Eco packaging plasmid. Plasmids were diluted in 1 mL of Opti-MEM™ Reduced Serum Medium (51985034, Gibco). Separately, 40 μL of TransIT-LT1 was diluted in 0.5 mL of Opti-MEM and incubated for 5 minutes. The diluted DNA and TransIT-LT1 mixtures were combined and gently mixed, then incubated for 20 minutes at room temperature to allow complex formation. During this incubation, the cell culture medium was replaced with fresh complete DMEM. The transfection complex was then added dropwise to the cell monolayer to ensure even distribution. Cells were returned to the incubator and maintained at 37 °C with 5% CO_2_. Retroviral supernatants were harvested 30–40 hours post-transfection, centrifuged at 400 × g for 5 minutes to remove cellular debris, and filtered through a 0.45 μm PVDF syringe filter (Millex-HV, Millipore, USA) under sterile conditions. Viral supernatants were either used immediately for T cell transduction.

### CAR T cell generation and basal ATG5 expression analysis

Following 24-hour activation, murine CD8^+^ T cells were transduced with two retroviral vectors encoding the human CD19-specific CAR (hCD19 CAR) and either ATG5 or LC3b OE constructs (**Supplementary Figure 1A**). An empty retroviral vector (pMIG) lacking any additional transgene was used as a control to generate CAR-T cells expressing the identical hCD19 CAR without autophagy-related gene OE (**Supplementary Figure 1A**). Cells were seeded into non-treated 6-well plates (SPL Life Sciences) at 1–3 × 10^6^ cells/well in 2 mL of medium. Cells were seeded into non-treated 6-well plates (SPL Life Sciences) at 1–3 × 10^6^ cells/well in 2 mL of medium. For retroviral transduction, 4 mL of viral supernatant (2 mL hCD19 CAR + 2 mL OE construct) was supplemented with 15 μg/mL polybrene (TR-1003-G, Merck, Darmstadt, Germany), and the mixture was added to CD8^+^ T cells. Plates were centrifuged at 2,000 × g for 1 hour at 37 °C and incubated at 37 °C with 5% CO_2_. A second round of transduction was performed 24 hours later using the same procedure to enhance gene delivery efficiency. Transduced T cells were subsequently cultured in RPMI-1640 medium supplemented with 10% FBS, 1% penicillin-streptomycin (Gibco), 2 mM L-glutamine, 50 μM 2-mercaptoethanol, and 100 IU/mL IL-2. During expansion, cultures were scaled up daily by transferring cells to progressively larger flasks (25T, 75T) and doubling the culture volume with fresh medium supplementation to sustain proliferation. At day 5 post-activation, cells were washed twice with PBS, counted, assessed for viability by trypan blue exclusion, and resuspended for adoptive transfer or *in vitro* assays.

To assess basal ATG5 expression following CAR T cell generation, freshly generated mCAR-T cells were subjected to intracellular staining prior to tumor co-culture. Cells were fixed with 0.4% paraformaldehyde and permeabilized using the Foxp3/Transcription Factor Fixation/Permeabilization Kit (00-5521-00, eBioscience, Waltham, MA, USA) according to the manufacturer’s instructions. Permeabilized cells were incubated with a rabbit monoclonal anti-APG5L/ATG5 antibody (clone EPR4797; ab109490, Abcam, Cambridge, UK) diluted 1:200 in 1× permeabilization buffer for 60 min at 4 °C, followed by washing and incubation with donkey anti-rabbit IgG Alexa Fluor 647 (ab150075, Abcam) diluted 1:200 for 30 min at 4 °C in the dark. After staining, cells were washed and resuspended in FACS buffer for flow cytometric analysis.

### Autophagy flux analysis by flow cytometry

Autophagy flux was quantified using the Autophagy Assay Kit (ab270790, Abcam) according to the manufacturer’s instructions with minor modifications. CAR-T cells (pMIG control, ATG5 OE, and LC3b OE groups) were cultured under basal or autophagy-modulating conditions prior to staining.

For autophagy induction, cells were treated with rapamycin (20 μM) for 24 h. To inhibit autophagosome–lysosome fusion and lysosomal degradation, bafilomycin A1 (BafA1, 10 μM) was added for the final 3 h of incubation. Basal autophagy flux was assessed in parallel cultures treated with BafA1 alone or vehicle control.

Following treatments, cells were harvested and first subjected to surface and viability staining. Cells were incubated with Live/Dead fixable eFluor 780 viability (FVS eF780), anti-CD8 (BUV395), and anti-Thy1.1 (APC) antibodies for 15 min at 4 °C, protected from light. After surface staining, cells were washed with FACS buffer to remove unbound antibodies.

Subsequently, cells were resuspended at a density of 5 × 10^5^ cells/mL and stained with autophagy probe red. The probe was reconstituted and diluted according to the manufacturer’s instructions and added to the cell suspension at a final dilution of 1:50. Cells were incubated for 20 min at 37 °C protected from light, washed three times with FACS buffer, and resuspended in the same buffer for analysis.

Fluorescence was analyzed by flow cytometry using a PE-TexasRed. Data acquisition was gated on live CD8^+^ eGFP^+^ Thy1.1^+^ CAR-T cells. Autophagy flux was calculated as the difference in mean fluorescence intensity (ΔMFI) between BafA1-treated and corresponding untreated samples under basal or rapamycin-induced conditions. Flow cytometric data were analyzed using FlowJo software (v10.10.0; FlowJo LLC, Ashland, OR, USA).

### Western blotting assay

For Western blotting, cells were lysed in RIPA buffer (1% Triton X-100, 1% sodium deoxycholate, 0.1% SDS, 150 mM sodium chloride, 50 mM Tris-HCl, pH 7.4, 2 mM EDTA) supplemented with protease and phosphatase inhibitor cocktails. The supernatant was collected by centrifugation at 13,000 rpm for 20 minutes at 4 °C. Protein concentration was determined using the Pierce™ BCA Protein Assay Kit (23225, Thermo Fisher Scientific, Waltham, MA, USA). Equal amounts of protein were mixed with 4× Laemmli sample buffer (1610747, Bio-Rad, Hercules, CA, USA) containing 2-mercaptoethanol, boiled at 95 °C for 5 minutes, separated by 15% SDS-PAGE, and transferred onto a polyvinylidene difluoride (PVDF) membrane. Membranes were blocked with TBS-T containing 5% skim milk or BSA and incubated with anti-rabbit p62 (5114S, Cell Signaling Technology, Danvers, MA, USA), anti-rabbit LC3b (2775S, Cell Signaling Technology), or GAPDH (sc-32233, Santa Cruz, Dallas, TX, USA) primary antibodies overnight at 4 °C. After washing, the membranes were incubated with HRP-conjugated goat anti-rabbit (ADI-SAB-300-J, ENZO, Farmingdale, NY, USA) secondary antibodies for 1 hour at room temperature. Signals were detected using Immobilon Western Chemiluminescent HRP Substrate (WBKLS0500, EMD Millipore, Burlington, MA, USA) and visualized with the ImageQuant 800F imaging system (Cytiva, Marlborough, MA, USA).

### Real-time cytotoxicity assay

**Figure 1:**
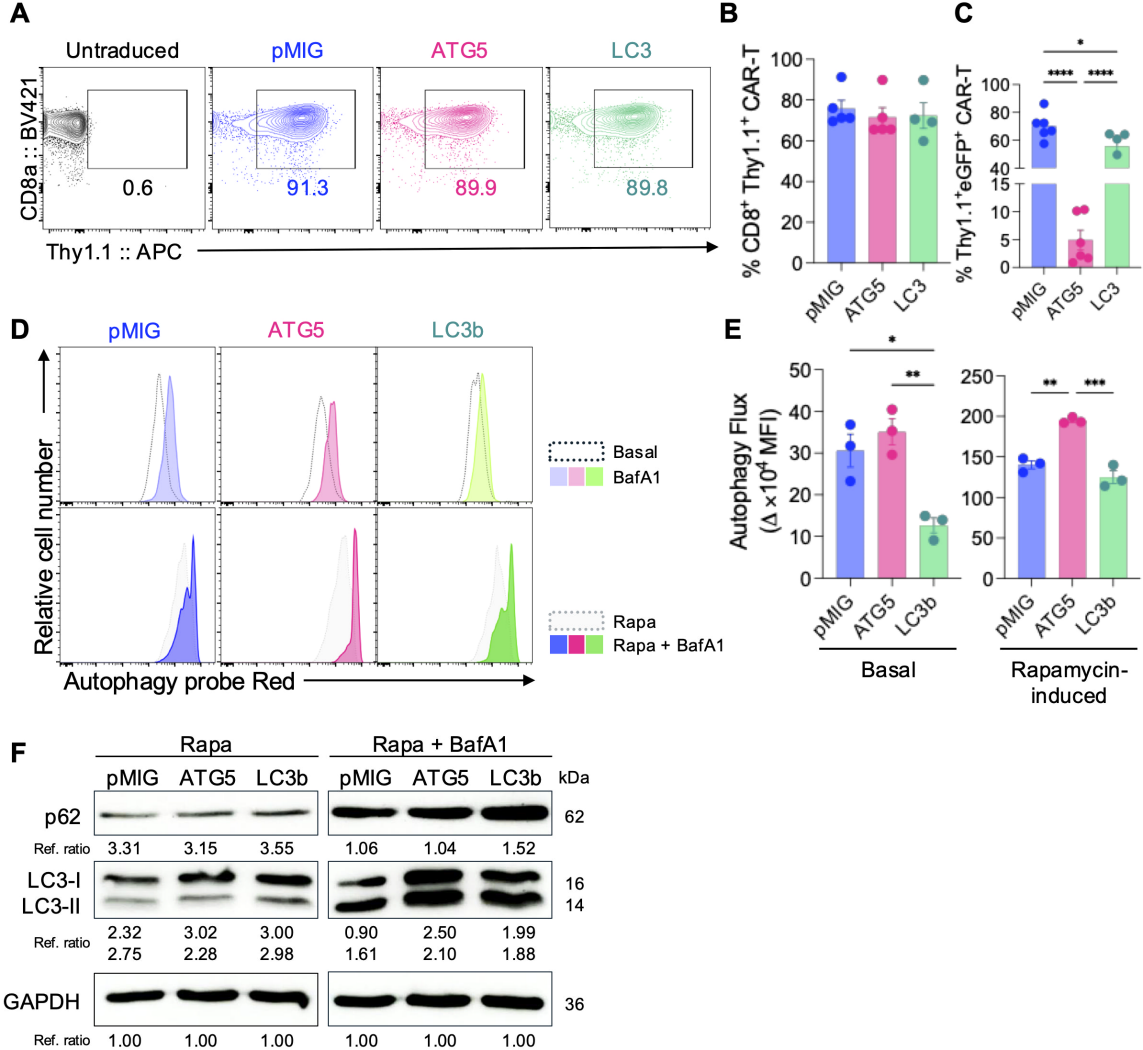
Characterization of ATG5 and LC3b OE mCAR-T cells and assessment of autophagy flux. **(A–C)** Flow cytometric analysis of CAR expression in CD8^+^ T cells transduced with pMIG (empty vector), ATG5 OE, or LC3b OE constructs. **(A)** Representative flow cytometry plots showing CD8α and Thy1.1 expression in untransduced CD8^+^ T cells and CAR-T cells transduced with pMIG control, ATG5, or LC3b constructs. **(B)** Quantification of CD8^+^Thy1.1^+^ CAR-T cell frequencies. **(C)** Quantification of eGFP^+^Thy1.1^+^ OE CAR-T cell frequencies. The schematic structures of the hCD19 CAR vector and OE vectors are shown in **Supplementary Figure 1A**, and the gating strategy is provided in **Supplementary Figure 1B**. **(D–F)** Assessment of autophagy flux. **(D)** Representative histograms of autophagosome staining using Autophagy Probe Red under basal conditions with or without bafilomycin A1 (BafA1) treatment (upper), and following rapamycin (Rapa) stimulation with or without BafA1 treatment (lower). **(E)** Quantification of autophagy flux expressed as mean fluorescence intensity (MFI) differences (ΔMFI ×10^4^) between conditions with and without BafA1 treatment under basal conditions (left) and rapamycin stimulation (right). **(F)** Representative immunoblot analysis of p62 and LC3 (LC3-I and LC3-II) in CAR-T cells treated with rapamycin alone or rapamycin plus BafA1. GAPDH was used as a loading control. Numbers below the blots indicate relative band intensities normalized to GAPDH. Individual replicates are shown as dots, and bars indicate mean ± SEM (n = 3). Statistical significance was determined using one-way ANOVA with Tukey’s *post hoc* test. *p < 0.05, **p < 0.01, ***p < 0.001, ****p < 0.0001.

To assess the cytolytic activity of CAR T cells *in vitro*, a real-time fluorescence-based killing assay was performed using the CellCyte X live-cell imaging system (**Figure 1D**; CytoSMART Technologies, Eindhoven, Netherlands). B16F0 murine melanoma cells stably expressing human CD19-mCherry (B16F0-hCD19-mCherry) were used as target cells. These cells were transduced in advance to express red fluorescent protein (RFP), enabling non-invasive monitoring of cell viability via fluorescence signal. Target cells were seeded at a density of 2 × 10^4^ cells per well into black-walled, clear-bottom 96-well plates (Corning, Corning, NY, USA) and allowed to adhere and stabilize for 24 hours at 37 °C with 5% CO_2_. Following this incubation, murine CAR T cells were added to the wells at varying effector-to-target (E:T) ratios, including 4:1, 2:1, 1:1, and 0.5:1, in triplicate for each condition. Control wells included target cells without effector cells to establish baseline fluorescence over time. After co-culture initiation, the plate was immediately transferred into the CellCyte X system, and live-cell images were captured every 6 hours for up to 96 hours. Imaging was conducted using the RFP channel to specifically detect B16F0-hCD19 cells, allowing for continuous tracking of target cell density and viability in the presence or absence of CAR T cells. B16F0-hCD19-mCherry confluency (%) were quantified using CytoSMART CellCyte analysis software. Time-lapse imaging also allowed visual confirmation of T cell-mediated killing dynamics.

For tumor re-challenge experiments, B16F0-hCD19-mCherry cells (0.1 × 10^6^ cells) were directly added to the same wells containing the ongoing CAR T cell–tumor cell co-culture at the indicated time point. Re-challenge was performed without transferring CAR T cells to new plates or pre-seeding tumor cells in separate wells, thereby maintaining the original co-culture environment.

### Tumor co-culture and flow cytometric analyses of ROS, effector function, and ATG5 expression

In this study, cytokine responses were primarily assessed by flow cytometry–based intracellular cytokine staining, which enables direct evaluation of CAR-T cell–intrinsic effector function at the single-cell level. In details, B16F0-hCD19 target cells were seeded at a density of 1 × 10^5^ cells per well and co-cultured with mCAR-T cells at an effector-to-target (E:T) ratio of 1:1. Co-cultures were maintained in TCM medium supplemented with recombinant human TGF-β (5 ng/mL; 100-21, PeproTech, Cranbury, NJ, USA) and IL-2 (100 IU/mL; 200-02, PeproTech) to mimic immunosuppressive TME conditions.

For intracellular cytokine staining (ICS), co-cultures were established in separate wells, and GolgiPlug™ (555029, BD Biosciences, San Jose, CA, USA) was added during the final 4 h incubation according to the manufacturer’s instructions. In parallel, independent wells without GolgiPlug™ were used for the assessment of intracellular and mitochondrial ROS as well as surface phenotypic marker expression.

After 24 h of co-culture, cells were harvested, washed with FACS buffer, and processed for flow cytometric analysis. Cellular ROS levels were measured using CellROX™ Deep Red (ab186029, Abcam), and mitochondrial ROS levels were assessed using MitoSOX™ Red (M36007, Thermo Fisher Scientific). For ROS staining, cells were first stained with Live/Dead Fixable eFluor™ 780 viability dye (FVS eF780), anti-CD8 (BUV395), and anti-Thy1.1 (APC) antibodies for 15 min at 4 °C. Cells were then washed with FACS buffer, followed by ROS staining with pre-warmed DPBS for 15 min at 37 °C protected from light. After washing with FACS buffer, cells were immediately analyzed by flow cytometry without fixation.

For ICS, cells were fixed with 0.4% paraformaldehyde and permeabilized using the Foxp3/Transcription Factor Fixation/Permeabilization Kit (00-5521-00, eBioscience) according to the manufacturer’s instructions. Intracellular cytokine and effector molecule staining was performed in 1× permeabilization buffer with antibodies diluted at 1:200 for 60 min at 4 °C. Following staining, cells were washed and resuspended in FACS buffer for flow cytometric analysis. Flow cytometric data were acquired using a Sony Cytek system (Cytek Biosciences, Fremont, CA, USA) and analyzed with FlowJo software (v10.10.0). Antibodies used for flow cytometry are listed in **Supplementary Table 2**.

### *In vivo* tumor model and CAR T cell transfer

To evaluate the antitumor efficacy of CAR T cells *in vivo*, a syngeneic murine tumor model was established using C57BL/6J mice (male, 6 weeks old). Mice were subcutaneously injected in the right flank with 3 × 10^5^ B16F0-hCD19 cells suspended in 100 μL of sterile phosphate-buffered saline (PBS). On day 6 post-tumor inoculation, mice received 5 Gy of total body IR to facilitate lymphodepletion. IR was delivered using an X-RAD 320 irradiator (Precision X-Ray, North Branford, CT, USA) with appropriate dosimetry calibration. The following day (day 7), mice were treated with 5–7.5 × 10^6^ CAR T cells suspended in 100 μL of sterile PBS, administered via retro-orbital intravenous injection under brief isoflurane anesthesia. CAR T cells were prepared fresh and washed extensively to remove residual cytokines and viral particles prior to injection. Tumor growth was monitored every 2–3 days using digital calipers. Tumor dimensions (length and width in mm) were recorded, and tumor area was calculated using the formula:

tumor area (mm²) = length × width.

Tumor growth was monitored until tumors reached 225 mm², which was defined as a humane endpoint in accordance with institutional animal care guidelines and commonly used criteria in solid tumor immunotherapy models.

### TIL isolation and flow cytometric analysis

TILs were isolated to evaluate immune cell infiltration and phenotype following CAR T cell therapy. Tumors were harvested from euthanized mice on day 20 post-tumor inoculation, corresponding to day 9 post-CAR T cell transfer (administered on day 12) and day 10 post-total body IR (administered on day 11; 5 Gy). Tumors were excised aseptically and transferred into gentleMACS C tubes (Miltenyi Biotec, Bergisch Gladbach, Germany) containing RPMI 1640 medium (Gibco) supplemented with Collagenase D (1 mg/mL; 11088866001, Roche, Basel, Switzerland) and DNase I (100 μg/mL; D5025-15KU, Sigma–Aldrich, St. Louis, MO, USA) Tissues were mechanically dissociated using the gentleMACS Dissociator (Miltenyi Biotec) with the preset program optimized for tumor digestion. After mechanical processing, tubes were incubated at 37 °C for 60 minutes in a shaking incubator set at 200 rpm to enzymatically digest the extracellular matrix and facilitate cell release. Following enzymatic digestion, the resulting tumor slurry was filtered through a 70 μm cell strainer (SPL Life Sciences) into 50 mL conical tubes to remove debris and undigested tissue fragments. The single-cell suspension was then centrifuged at 400 × g for 5 minutes at 4 °C and resuspended in RPMI 1640. To enrich for lymphocytes, cell suspensions were overlaid onto Lymphocyte Separation Medium (25-072-CV, Corning) and centrifuged at 400 × g for 20 minutes at room temperature without brake. The mononuclear cell layer at the interface was carefully collected using a Pasteur pipette, washed twice with PBS, and resuspended in FACS buffer (PBS + 2% FBS + 2 mM EDTA) for downstream immunophenotyping.

For flow cytometric immunophenotyping, isolated TILs were stained with fluorochrome-conjugated antibodies against surface markers. For intracellular cytokine analysis, TILs were stimulated ex vivo with Cell Stimulation Cocktail (00-4975-03, eBioscience) for 4 h at 37 °C, followed by fixation with 0.4% paraformaldehyde and permeabilization using the Foxp3/Transcription Factor Fixation/Permeabilization Kit (00-5521-00, eBioscience). Intracellular staining was performed in 1× permeabilization buffer with antibodies diluted at 1:200 for 30 min at 4 °C. For transcription factor analysis, TILs were processed without ex vivo stimulation, fixed and permeabilized using the same kit, and stained in 1× permeabilization buffer with antibodies diluted at 1:200 for 60 min at 4 °C. Flow cytometric data were acquired using a Sony Cytek system (Cytek Biosciences) and analyzed with FlowJo software (v10.10.0). Antibodies used for flow cytometry are listed in **Supplementary Table 2**.

### Statistical analysis

All statistical analyses were performed using Prism software (version 10.1.0; GraphPad, La Jolla, CA, USA). Data are expressed as mean ± SEM. Group comparisons were performed using one-way ANOVA followed by Tukey’s *post hoc* test. Two-group comparisons were analyzed using an unpaired, two-tailed Student’s t-test. Statistical significance is indicated by asterisks as defined in the figure legends.

## Results

### Characterization of ATG5 and LC3b OE CAR-T cells and assessment of autophagy flux

To evaluate how OE of autophagy-related genes influences the basal properties and autophagic regulation of CAR-T cells, murine CD8^+^ T cells were transduced with CAR vectors encoding either pMIG control, ATG5 or LC3b OE (**Supplementary Figure 1A**). Flow cytometric analysis showed that the overall frequency of CD8^+^Thy1.1^+^ CAR-T cells did not differ significantly among the three groups, indicating comparable CAR transduction efficiencies (**Figures 1A, B**).

In contrast, the proportion of eGFP^+^Thy1.1^+^ OE CAR-T cells differed between groups, with ATG5 OE CAR-T cells exhibiting a significantly lower fraction compared with pMIG and LC3b OE CAR-T cells (**Figure 1C**). This suggests that co-expression efficiency of the ATG5 construct may be relatively limited. Nevertheless, ATG5 protein expression was significantly elevated in ATG5 OE CAR-T cells compared with the control group (**Supplementary Figure 1B**). All subsequent functional analyses were therefore conducted using gated eGFP^+^Thy1.1^+^ CAR-T cell populations to ensure consistency across groups (**Supplementary Figure 1C**).

To determine whether ATG5 or LC3b OE alters autophagic activity in CAR-T cells, flow cytometric analysis was performed using an autophagosome-specific fluorescent probe. Under basal conditions, ATG5 OE alone did not result in a significant increase in autophagy flux, while LC3b OE CAR-T cells displayed a comparatively reduced basal autophagy flux relative to pMIG controls (**Figures 1D, E**). These findings indicate that OE of autophagy-related genes does not necessarily enhance autophagy under unstressed conditions.

By contrast, pharmacological stress induced by mTOR inhibition using rapamycin led to increased autophagy probe signals in all groups (20, 21). Notably, ATG5 OE CAR-T cells exhibited a markedly greater increase in autophagy flux compared with both pMIG and LC3b OE groups, indicating an enhanced inducible autophagic response under stress conditions (**Figures 1D, E**). Thus, ATG5 OE appears to augment the capacity to mobilize autophagy in response to stress rather than constitutively activating autophagy at baseline.

This trend was corroborated by western blot analysis (**Figure 1F**). Under combined rapamycin and bafilomycin A1 treatment, ATG5 OE CAR-T cells showed increased accumulation of LC3-II (approximately 1.5-fold) compared with pMIG controls, whereas p62 levels were not significantly altered. These results suggest that ATG5 OE enhances the potential for autophagosome formation and LC3 lipidation during mTOR-dependent autophagy induction, while changes in cargo degradation remain less evident. This interpretation is consistent with flow cytometric data showing a greater rapamycin-induced ΔMFI of the autophagy probe in ATG5 OE CAR-T cells relative to pMIG controls, supporting enhanced inducible autophagic responsiveness. In contrast, LC3b OE CAR-T cells displayed p62 accumulation alongside limited autophagy flux by FACS, suggesting that LC3b OE alone does not effectively translate into improved functional autophagy turnover.

Collectively, these findings demonstrate that ATG5 OE does not constitutively elevate basal autophagy flux in CAR-T cells but instead increases the inducible capacity to engage autophagy under mTOR-inhibitory stress conditions. In contrast, LC3b OE shows limited association with enhanced inducible autophagy or improved autophagic turnover in CAR-T cells.

### *In vitro* antitumor activity of ATG5 and LC3b OE CAR-T cells under TME-mimicking conditions

**Figure 2:**
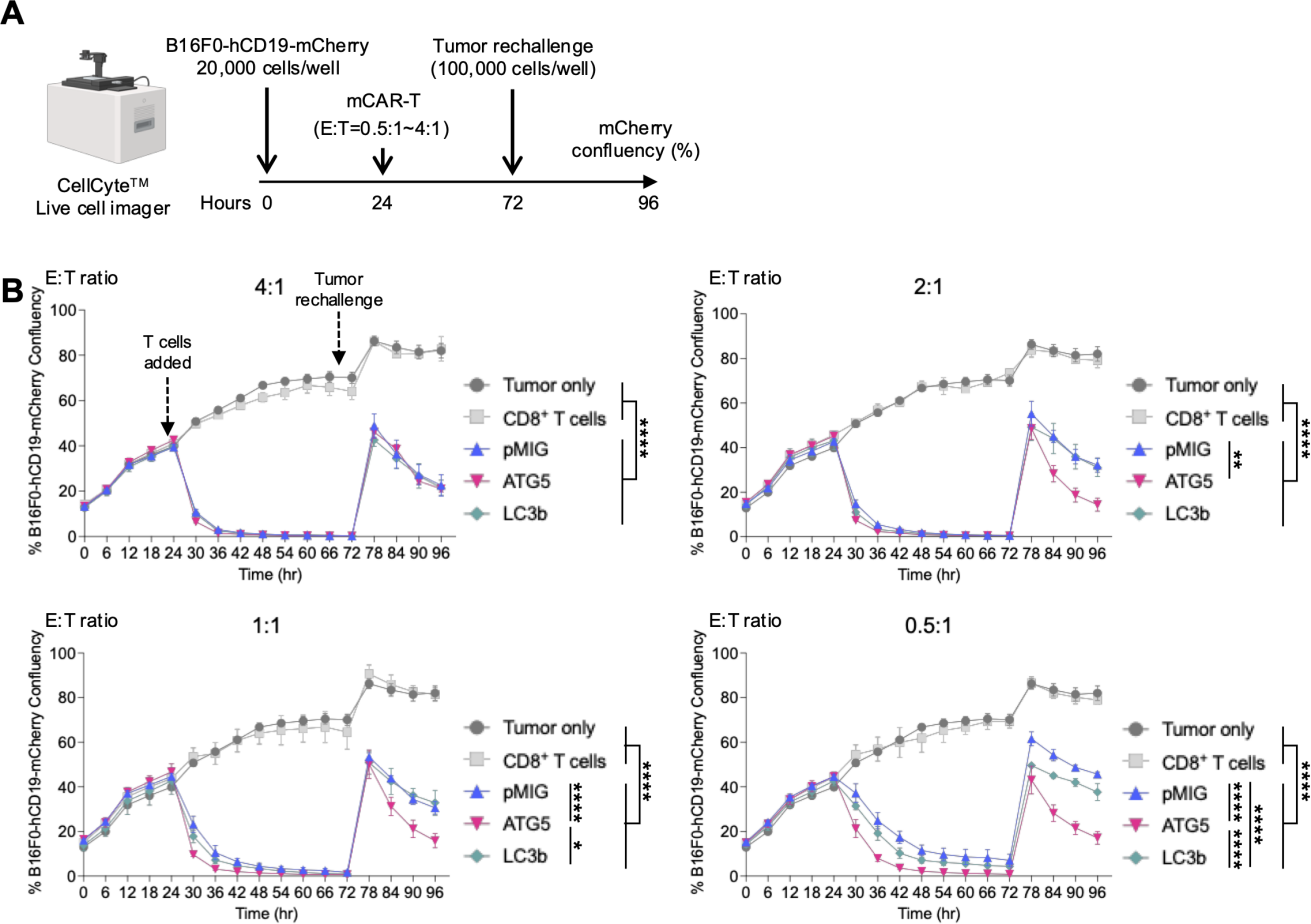
*In vitro* cytotoxicity of ATG5 or LC3b OE mCAR-T cells under an immunosuppressive TME–mimicking condition. **(A)** Schematic overview of the *in vitro* long-term cytotoxicity assay. mCAR-T cells were co-cultured with B16F0-hCD19-mCherry tumor cells at various effector-to-target (E:T) ratios (0.5:1 to 4:1) for 96 h in the presence of TGF-β (5 ng/mL) to mimic an immunosuppressive TME. Tumor cell confluency was quantified based on mCherry fluorescence intensity using a CellCyte™ live-cell imaging system. **(B)** Cytotoxicity kinetics of each mCAR-T cell group over time across different E:T ratios. Data represent mean ± SEM (n = 3). Statistical analysis was performed using two-way ANOVA followed by Tukey’s *post hoc* test to compare the main effects among pMIG, ATG5 OE, and LC3b OE CAR-T cells within each E:T ratio. *p < 0.05; **p < 0.01; ****p < 0.0001.

To determine whether the enhanced inducible autophagy observed in ATG5 OE CAR-T cells (**Figure 1**) translates into improved antitumor function, we evaluated CAR-T cytotoxicity under immunosuppressive conditions mimicking the TME. mCAR-T cells were co-cultured long-term with B16F0-hCD19 tumor cells in the presence of TGF-β at various effector-to-target (E:T) ratios, and cytotoxic durability was assessed in a tumor rechallenge setting (**Figure 2A**).

During the initial phase of co-culture, pMIG, ATG5 OE, and LC3b OE CAR-T cells exhibited distinct tumor suppression profiles depending on the E:T ratio. At a relatively high E:T ratio (4:1), no substantial differences in early tumor control were observed among the groups. However, at intermediate E:T ratios (2:1 and 1:1), ATG5 OE CAR-T cells consistently demonstrated modest but reproducible improvements in early tumor suppression compared with both pMIG and LC3b OE CAR-T cells. This trend became more pronounced at the lowest E:T ratio (0.5:1), where ATG5 OE CAR-T cells exhibited the strongest initial antitumor activity among the three groups (**Figure 2B**).

Differences in cytotoxic durability became more evident during prolonged co-culture with tumor rechallenge (after 72 h). At a high E:T ratio (4:1), ATG5 OE CAR-T cells did not show a statistically significant advantage over pMIG or LC3b OE CAR-T cells in sustained tumor control. In contrast, at E:T ratios of 2:1 and 1:1, ATG5 OE CAR-T cells maintained tumor suppression more stably than either pMIG or LC3b OE CAR-T cells. Even under the lowest E:T ratio (0.5:1), ATG5 OE CAR-T cells preserved relatively high levels of cytotoxicity over time (**Figure 2B**). LC3b OE CAR-T cells displayed limited improvement over pMIG controls under certain conditions but consistently exhibited inferior cytotoxic durability compared with ATG5 OE CAR-T cells.

Collectively, these results indicate that the functional benefit conferred by ATG5 OE is most apparent under conditions of limited effector cell availability and repeated tumor stimulation, rather than under conditions of abundant effector cell excess. This suggests that ATG5-mediated enhancement of inducible autophagy preferentially supports sustained CAR-T cell function in immunosuppressive, resource-constrained environments that more closely resemble the TME.

### ATG5 OE attenuates oxidative stress and enhances effector-associated responses under TME-mimicking conditions

**Figure 3:**
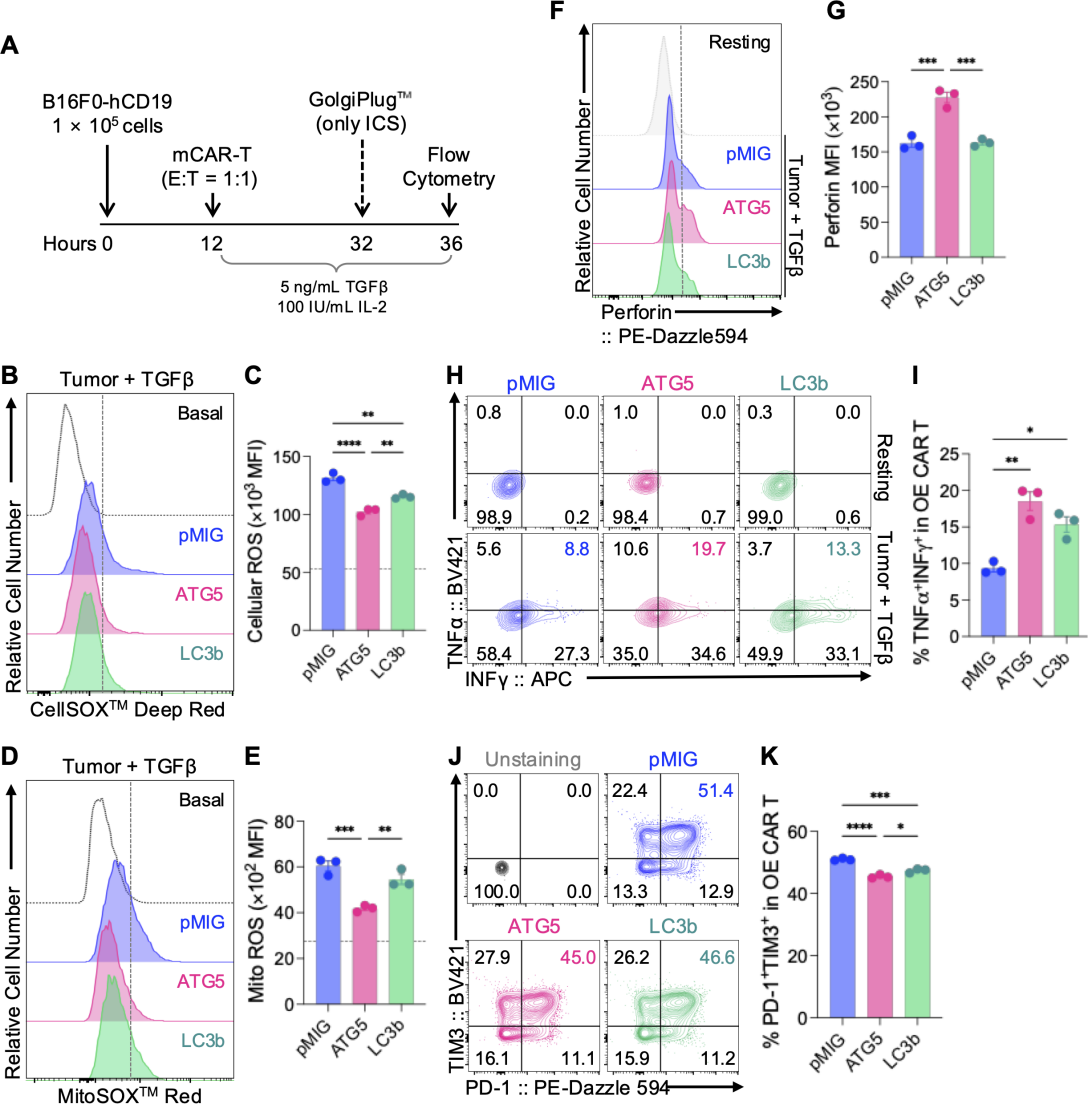
ATG5 OE reduces oxidative stress while enhancing effector function of mCAR-T cells under TGF-β–mediated immunosuppressive conditions. **(A)** Schematic illustration of the experimental design for assessing oxidative stress and effector functional characteristics. B16F0-hCD19 target cells (1 × 10^5^ cells) were co-cultured with mCAR-T cells at an effector-to-target (E:T) ratio of 1:1 in the presence of TGF-β (5 ng/mL) and IL-2 (100 IU/mL). Intracellular cytokine staining (ICS) assays were performed in separate wells supplemented with GolgiPlug™, whereas ROS measurements and phenotypic analyses were conducted in parallel wells without GolgiPlug™. After 24 h of co-culture, cells were harvested and subjected to flow cytometric analysis. **(B–E)** Assessment of intracellular and mitochondrial ROS in mCAR-T cells co-cultured with B16F0-hCD19 tumor cells in the presence of TGF-β (5 ng/mL). **(B)** Representative histograms of total cellular ROS measured by CellROX™ Deep Red staining. **(C)** Quantification of cellular ROS levels expressed as mean fluorescence intensity (MFI ×10³). **(D)** Representative histograms of mitochondrial superoxide measured by MitoSOX™ Red staining. **(E)** Quantification of mitochondrial ROS levels expressed as MFI (×10²). In panels **(C, E)**, gray dotted lines indicate the basal status without tumor co-culture. **(F–I)** Functional characterization of mCAR-T cells following co-culture with B16F0-hCD19 tumor cells under TGF-β–mediated immunosuppressive conditions. **(F)** Representative histograms of intracellular perforin staining. **(G)** Quantification of perforin expression expressed as MFI (×10³). **(H)** Representative flow cytometry plots showing IFN-γ and TNF-α expression in pMIG, ATG5 OE, and LC3b OE mCAR-T cells. **(I)** Quantification of TNF-α^+^IFN-γ^+^ cells among OE mCAR-T cells. **(J, K)** Analysis of exhaustion marker expression. **(J)** Representative flow cytometry plots showing PD-1 and TIM-3 expression in OE mCAR-T cells. **(K)** Quantification of PD-1^+^TIM-3^+^ cells among OE mCAR-T cells. Individual replicates are shown as dots, and bars indicate mean ± SEM (n = 3). Statistical significance was determined using one-way ANOVA with Tukey’s *post hoc* test. *p < 0.05, **p < 0.01, ***p < 0.001, ****p < 0.0001. See also **Supplementary Figure 2**.

To investigate the mechanisms underlying the enhanced *in vitro* antitumor activity of ATG5 OE CAR-T cells, we analyzed oxidative stress levels as well as effector function– and phenotype-associated parameters under immunosuppressive conditions mimicking the TME in the presence of TGF-β (**Figure 3A**).

Under co-culture conditions with B16F0-hCD19 tumor cells and TGF-β treatment, ATG5 OE CAR-T cells exhibited significantly reduced levels of both total cellular ROS and mitochondrial ROS compared with pMIG and LC3b OE CAR-T cells (**Figures 3B–E**). These findings indicate that ATG5 OE contributes to the mitigation of oxidative stress in CAR-T cells exposed to TGF-β–mediated stress.

Concomitant with the reduction in oxidative stress, ATG5 OE CAR-T cells showed enhanced expression of effector-associated molecules. Intracellular staining revealed significantly higher perforin expression in ATG5 OE CAR-T cells compared with both pMIG and LC3b OE CAR-T cells (**Figures 3F, G**). In addition, the frequency of IFN-γ^+^TNF-α^+^ cells was highest in the ATG5 OE group among all conditions examined (**Figures 3H, I**). These results suggest that ATG5 OE supports the maintenance or enhancement of effector molecule production and cytokine secretion capacity in CAR-T cells, even under immunosuppressive conditions.

In contrast, changes in phenotypes associated with exhaustion and differentiation were generally modest. The exhaustion phenotype assessed by co-expression of PD-1 and TIM-3 showed a slight reduction in ATG5 OE CAR-T cells compared with pMIG controls; however, the magnitude of this difference was limited (**Figures 3J, K**). Moreover, no significant differences were observed among groups in the frequency of TCF1^+^TIM-3^-^ cells, Ki67 expression, PD-1^+^TOX^+^ cells, or KLRG1^+^PD-1^+^ and KLRG1^+^TIM-3^+^ populations (**Supplementary Figure 2**).

Taken together, the enhanced *in vitro* antitumor activity associated with ATG5 OE appears to be linked more closely to attenuation of oxidative stress and the accompanying enhancement of effector molecule expression and cytokine secretion under TGF-β–mediated immunosuppressive conditions, rather than to overt changes in memory differentiation or exhaustion phenotypes. These intracellular functional adaptations provide a mechanistic basis for the improved tumor control observed in ATG5 OE CAR-T cells. Overall, LC3B OE showed limited association with enhanced autophagy flux or functional benefit in CAR-T cells, while ATG5 OE exhibited a clearer relationship with inducible autophagy and functional persistence under stress conditions. On this basis, subsequent analyses and *in vivo* studies were centered on ATG5.

### ATG5 OE mCAR-T cells exhibit enhanced antitumor efficacy *in vivo* under IR-preconditioned conditions

**Figure 4:**
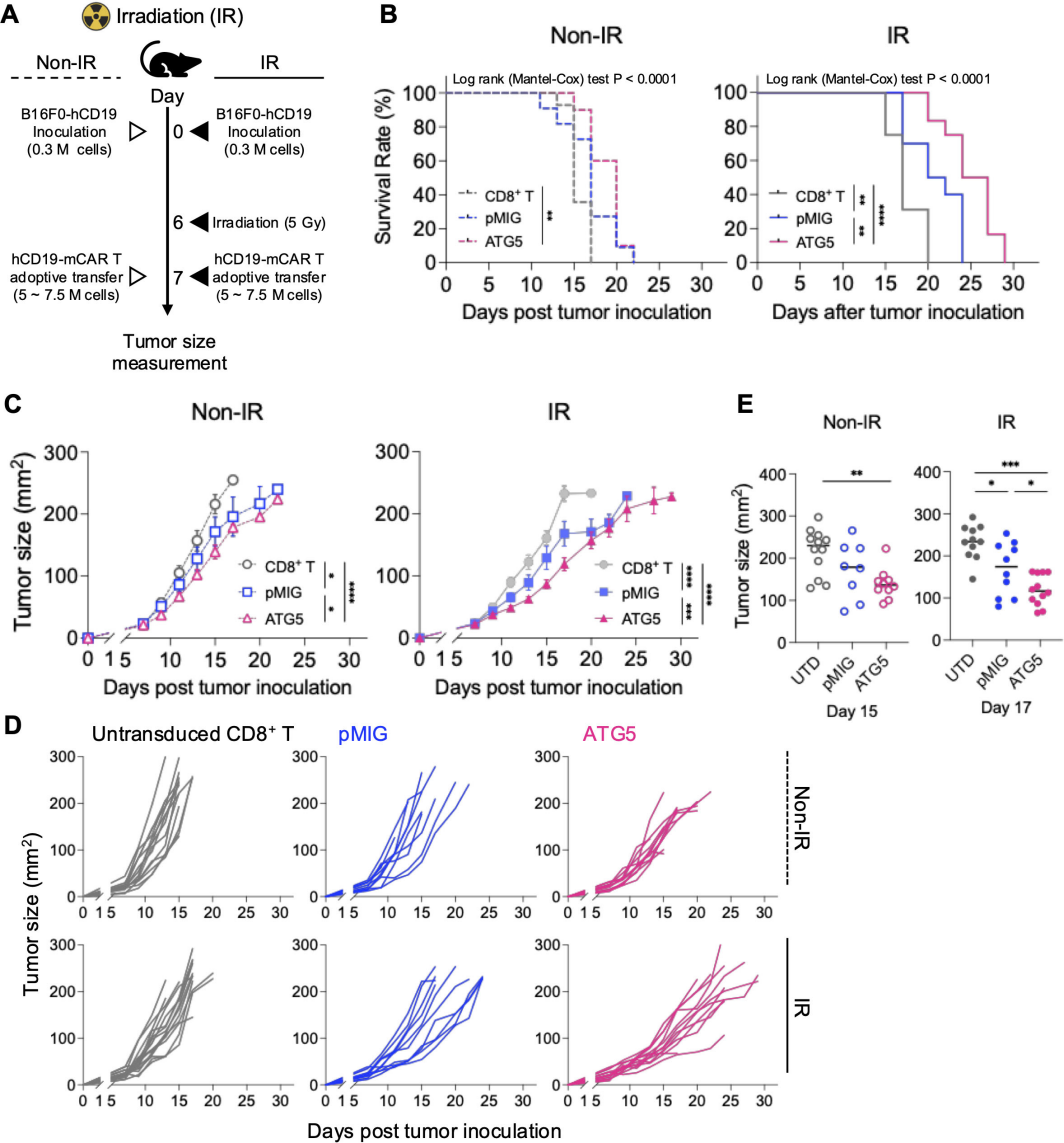
ATG5 OE mCAR-T cells exhibit enhanced *in vivo* antitumor efficacy with irradiation preconditioning. **(A)** Schematic overview of the *in vivo* experimental design. C57BL/6 mice were subcutaneously inoculated with B16F0-hCD19 tumor cells (3 × 10^5^ cells). In the IR group, whole-body irradiation (5 Gy) was administered on day 6, followed by adoptive transfer of hCD19 mCAR-T cells (5–7.5 × 10^6^ cells) on day 7. In the Non-IR group, mCAR-T cells were adoptively transferred without irradiation. Tumor size was monitored at the indicated time points. **(B)** Kaplan–Meier survival analysis. Survival curves of tumor-bearing mice treated with untransduced CD8^+^ T cells (UTD), pMIG control, or ATG5 OE mCAR-T cells under Non-IR (left, dashed lines) or IR (right, solid lines) conditions. A tumor size ≥ 225 mm² was used as a surrogate endpoint for survival analysis for humane reasons. Statistical significance was assessed using the log-rank (Mantel-Cox) test, followed by Holm–Šídák’s multiple comparisons test for group comparisons. **(C)** Tumor growth kinetics. Mean tumor growth curves under Non-IR (left) and IR (right) conditions. Data are presented as mean ± SEM. Statistical significance was determined using two-way ANOVA with Tukey’s *post hoc* test. **(D)** Individual tumor growth trajectories. Tumor growth curves for individual mice treated with UTD, pMIG, or ATG5 OE mCAR-T cells under Non-IR (upper panels) and IR (lower panels) conditions. Group sizes were as follows: Non-IR, n = 15, 10, and 11; IR, n = 16, 10, and 12 for UTD CD8^+^ T cells, pMIG, and ATG5 OE mCAR-T cells, respectively. **(E)** Comparison of tumor sizes at defined time points. Tumor sizes measured on day 15 (Non-IR) and day 17 (IR). Each dot represents an individual mouse, and horizontal lines indicate mean values. Data are presented as mean ± SEM. Group sizes were as follows: Non-IR, n = 12, 8, and 10; IR, n = 11, 10, and 12 for UTD CD8^+^ T cells, pMIG, and ATG5 OE mCAR-T cells, respectively. Statistical significance was determined using one-way ANOVA with Tukey’s *post hoc* test. *p < 0.05, **p < 0.01, ***p < 0.001, ***p < 0.0001..

To evaluate the *in vivo* antitumor efficacy of ATG5 OE mCAR-T cells, tumor growth and survival were compared under conditions with or without IR preconditioning (**Figure 4A**). A syngeneic B16F0-hCD19 melanoma model was used, in which CAR-T cells targeting human CD19 were administered via retro-orbital intravenous injection, as described in the Methods. IR preconditioning provides a permissive environment that facilitates CAR-T cell access to the tumor and early engraftment, whereas the Non-IR setting represents a more restrictive condition in which these advantages are limited (22–24).

Under Non-IR conditions, treatment with ATG5 OE mCAR-T cells resulted in a modest reduction in tumor growth compared with pMIG control mCAR-T cells; however, overall tumor control and survival benefits remained limited (**Figures 4B–E**). In contrast, under IR-preconditioned conditions, mice treated with ATG5 OE mCAR-T cells exhibited a pronounced improvement in survival relative to both pMIG mCAR-T–treated and untransduced CD8^+^ T cell–treated groups (**Figure 4B**). Consistently, tumor growth was significantly suppressed in the ATG5 OE mCAR-T cell group under IR conditions (**Figures 4C, D**). Comparison of tumor burden at defined time points further demonstrated that ATG5 OE mCAR-T cell–treated mice maintained the smallest tumor volumes under IR-preconditioned conditions (**Figure 4E**).

These findings suggest that ATG5 OE does not primarily enhance initial tumor access or engraftment of CAR-T cells, but rather strengthens their ability to sustain antitumor activity after entering the TME, even under immunosuppressive conditions. Accordingly, ATG5 OE appears to function as an amplifier of CAR-T cell efficacy in settings where tumor accessibility has been secured by IR, thereby enabling more durable antitumor responses *in vivo*.

### *In vivo* TIL analysis reveals a functional basis for the antitumor activity of ATG5 OE CAR-T cells

**Figure 5:**
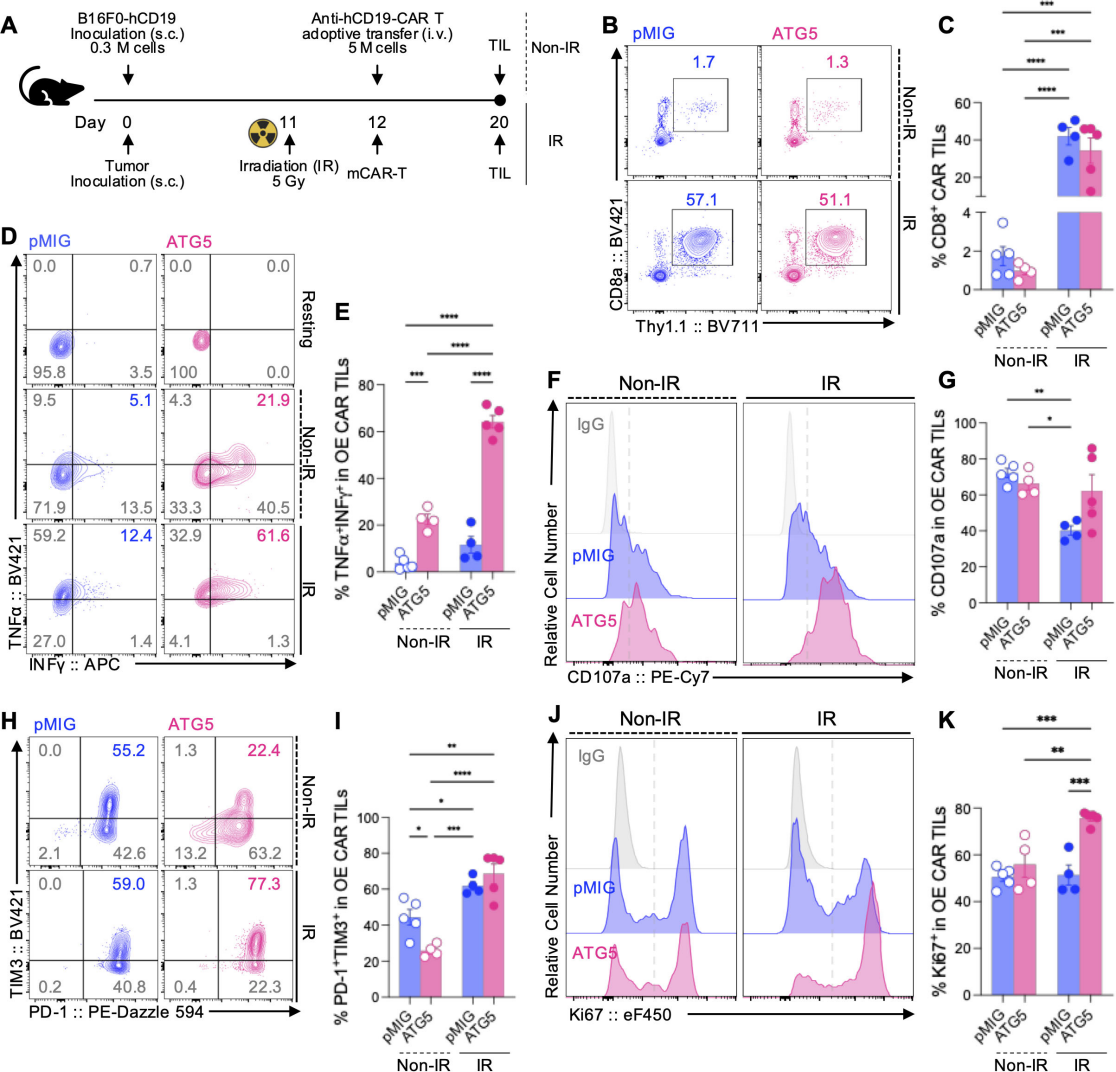
ATG5 OE enhances the functional activity of mCAR-T cells *in vivo* under IR conditions. **(A)** Schematic overview of the *in vivo* tumor model and TIL analysis. C57BL/6 mice were subcutaneously inoculated with B16F0-hCD19 tumor cells (3 × 10^5^ cells). In the IR group, mice received IR (5 Gy) on day 11, followed by adoptive transfer of anti-hCD19 mCAR-T cells (5 × 10^6^ cells, i.v.) on day 12. In the Non-IR group, mCAR-T cells were transferred without IR. Tumors were harvested on day 20 for TIL analysis. **(B, C)** Tumor infiltration of CAR-T cells under Non-IR and IR conditions. **(B)** Representative flow cytometry plots showing CD8α and Thy1.1 expression among TILs under Non-IR (upper) and IR (lower) conditions. **(C)** Quantification of CD8^+^ CAR-T cells among total TILs. **(D, E)** Cytokine-producing capacity of CD8^+^ OE mCAR-TILs. The gating strategy is shown in **Supplementary Figure 3A**. **(D)** Representative flow cytometry plots showing IFN-γ and TNF-α expression. **(E)** Quantification of TNF-α^+^IFN-γ^+^ cells among CD8^+^ OE mCAR-TILs. **(F, G)** Degranulation capacity of CD8^+^ OE mCAR-TILs. **(F)** Representative histograms of CD107a expression (IgG shown as a control). **(G)** Quantification of CD107a^+^ cells among CD8^+^ OE mCAR-TILs. **(H, I)** Expression of exhaustion markers on CD8^+^ OE mCAR-TILs. **(H)** Representative flow cytometry plots showing PD-1 and TIM-3 expression. **(I)** Quantification of PD-1^+^TIM-3^+^ cells among CD8^+^ OE mCAR-TILs. **(J, K)** Proliferative status of CD8^+^ OE mCAR-TILs. **(J)** Representative histograms of Ki67 expression (IgG shown as a control). **(K)** Quantification of Ki67^+^ cells among CD8^+^ OE mCAR-TILs. Individual replicates are shown as dots, and bars indicate mean ± SEM. Open dots represent the Non-IR group, and filled dots represent the IR group. Statistical significance was determined using one-way ANOVA with Tukey’s *post hoc* test. *p < 0.05, **p < 0.01, ***p < 0.001, ****p < 0.0001. See also **Supplementary Figure 3**.

To determine whether the therapeutic benefit of ATG5 OE CAR-T cells observed *in vivo* (**Figure 4**) is attributable to enhanced tumor infiltration or to sustained functional activity within the immunosuppressive TME, TILs were analyzed under IR and Non-IR conditions (**Figure 5A**) (25, 26).

Assessment of CAR-T cell infiltration revealed that, under Non-IR conditions, intratumoral accumulation of CD8^+^ CAR-T cells was limited in both pMIG and ATG5 OE groups, with no significant difference (**Figures 5B, C**). Under IR-preconditioned conditions, overall infiltration of CD8^+^ CAR-T cells was markedly increased; however, ATG5 OE CAR-T cells did not exhibit a substantially higher infiltration rate compared with pMIG controls. These results indicate that ATG5 OE does not directly enhance the tumor infiltration capacity of CAR-T cells.

In contrast, functional activation of tumor-infiltrating CD8^+^ OE CAR-T cells was selectively enhanced by ATG5 OE under IR conditions. Consistent with *in vitro* observations, the proportion of CD8^+^ OE CAR-T cells within the tumor was lower in the ATG5 OE group than in pMIG controls (**Supplementary Figure 3B**). To enable a fair functional comparison following tumor entry, all subsequent TIL analyses were therefore performed using gated CD8^+^ OE CAR-T cell populations (**Supplementary Figure 3A**).

Within this gated population, ATG5 OE CAR-TILs under IR conditions exhibited a significantly higher frequency of IFN-γ^+^TNF-α^+^ cells compared with pMIG controls (**Figures 5D, E**), along with sustained elevation of CD107a expression (**Figures 5F, G**). While these differences were less apparent under Non-IR conditions, they became pronounced following IR preconditioning, indicating that ATG5 OE preferentially enhances CAR-T cell effector function within the stressed tumor milieu.

Comprehensive analysis of exhaustion- and differentiation-associated phenotypes further suggested that the antitumor effect of ATG5 OE is not driven by selective expansion of specific differentiation subsets or reinforcement of stem-like characteristics. Under IR conditions, no significant difference was observed between ATG5 OE and pMIG CAR-TILs in the PD-1^+^TIM-3^+^ population (**Figures 5H, I**). Additional analyses revealed a modest increase in PD-1^+^TOX^+^ cells in ATG5 OE CAR-TILs, whereas no significant expansion of the TCF1^+^TIM-3^-^ stem-like population was detected (**Supplementary Figures 3C–F**). Likewise, central memory-like and effector memory-like subsets were not markedly restructured by ATG5 OE (**Supplementary Figures 3G, H**).

Taken together, comparative analysis of IR and Non-IR conditions indicates that the benefit conferred by ATG5 OE is not mediated by increased tumor infiltration, but rather by enhanced intracellular functional adaptation that allows CAR-T cells to maintain effector activity after tumor entry under immunosuppressive TME conditions. These *in vivo* TIL findings provide functional support for the superior antitumor efficacy of ATG5 OE CAR-T cells observed under irradiation-preconditioned settings.

## Discussion

This study was designed to determine whether ATG5-mediated autophagy regulation contributes to the functional persistence of CAR-T cells under solid tumor–associated stress conditions. We specifically examined how modulation of autophagy influences the stability of effector function when CAR-T cells are exposed to sustained immunosuppressive cues within the TME.

At the mechanistic level, ATG5 OE did not lead to a constitutive increase in basal autophagy activity. Instead, ATG5 OE CAR-T cells selectively exhibited enhanced autophagy flux under autophagy-inducing conditions, as evidenced by increased autophagosome accumulation upon autophagy induction and lysosomal blockade (**Figures 1D–F**). This pattern indicates that ATG5 OE increases the capacity of CAR-T cells to activate an autophagic response when challenged, rather than enforcing a persistently elevated autophagy state. Such inducible regulation is consistent with the established role of autophagy as a context-dependent stress adaptive process rather than a continuously active effector mechanism (27).

Enhanced inducible autophagy flux in ATG5 OE CAR-T cells was associated with reduced accumulation of cellular and mitochondrial ROS under TME-mimicking immune suppressive condition (**Figures 3B–E**). Excessive ROS generation is a well-recognized driver of mitochondrial damage, T cell dysfunction, and exhaustion within the TME (28–30). he observed reduction in oxidative stress therefore suggests that ATG5-mediated autophagy regulation contributes to intracellular redox homeostasis during immunesuppressive conditions. By facilitating the clearance of damaged cellular components and limiting ROS accumulation, inducible autophagy may support the maintenance of effector competence under tumor-associated stress, in agreement with previous studies highlighting the importance of redox control in preserving T cell functionality (31, 32).

Consistent with this interpretation, ATG5 OE CAR-T cells demonstrated improved functional durability *in vitro* under tumor-mimicking immunosuppressive conditions. During prolonged co-culture with tumor cells, ATG5 OE CAR-T cells maintained cytotoxic activity more stably over time compared with pMIG control cells (**Figure 2B**). This functional stability was accompanied by sustained effector cytokine responses (**Figures 3F–I**), indicating that ATG5-mediated autophagy regulation supports the maintenance of effector competence. Collectively, these findings suggest that ATG5 OE confers a functional advantage not by transiently amplifying CAR-T cell activation, but by delaying the functional collapse of effector programs under sustained immunosuppressive stimulation.

To determine whether this functional advantage reflected general accumulation of autophagy-related proteins or specific regulation of autophagy flux, we directly compared ATG5 OE with LC3b OE, which targets a downstream structural component of the autophagosome (19). Although LC3b OE CAR-T cells showed increased autophagosome-associated signals, this did not translate into a coordinated enhancement of inducible autophagy flux (**Figures 1D–F**). In contrast to ATG5 OE CAR-T cells, LC3b OE was associated with reduced functional stability and less effective control of cellular and mitochondrial ROS under immunosuppressive tumor-mimicking conditions (**Figures 3B–I**). Accordingly, LC3b OE CAR-T cells showed only limited improvement in antitumor activity *in vitro* relative to ATG5 OE CAR-T cells (**Figure 2**). These results indicate that functional support of CAR-T cells depends on the regulation of inducible autophagy capacity and associated stress-buffering responses, rather than autophagosome accumulation per se, highlighting a mechanistic distinction between ATG5- and LC3b-mediated autophagy modulation (33–36). On this basis, subsequent *in vivo* analyses were focused exclusively on ATG5 OE CAR-T cells.

*In vivo* analyses revealed that the functional consequences of ATG5 OE were strongly influenced by tumor accessibility. Under Non-IR conditions, both pMIG and ATG5 OE CAR-T cells exhibited markedly limited intratumoral accumulation (**Figures 5B, C**). In this context, ATG5 OE CAR-T cells did not display significant increases in degranulation (CD107a) or proliferation (Ki-67), indicating that insufficient tumor access constrained full effector engagement. Consequently, the observed reductions in exhaustion-associated markers under Non-IR conditions should be interpreted cautiously, as limited antigen exposure itself may attenuate chronic stimulation rather than reflect intrinsic enhancement of effector function. Consistent with this interpretation, the condition-dependent patterns observed in PD-1^+^TOX^+^ and PD-1^+^TIM-3^+^ CAR-T cells (**Figures 5H, I**; **Supplementary Figures 3C, D**) are more likely attributable to differences in tumor accessibility and antigen exposure than to opposing exhaustion states.

Despite these environmental constraints, ATG5 OE CAR-T cells under Non-IR conditions exhibited increased IFN-γ and TNF-α production without corresponding increases in CD107a or Ki-67 expression (**Figures 5D–G**, **5J, K**). This dissociation suggests that ATG5-mediated autophagy regulation preserves cytokine-producing capacity even when sustained cytotoxic execution or proliferative expansion is limited. Such partial functional preservation may reflect reduced oxidative stress at the intracellular level, rather than enhanced tumor engagement per se. However, the absence of increased degranulation or proliferation further indicates that a role for ATG5 in maintaining intracellular functional integrity rather than overcoming extrinsic barriers to tumor access.

By contrast, under IR-preconditioned conditions—where tumor accessibility is improved—ATG5-mediated functional advantages were translated into measurable antitumor efficacy. ATG5 OE CAR-T cells exhibited significantly enhanced tumor control relative to control group (**Figures 4B–E**), accompanied by increased intratumoral effector cytokine production (**Figures 5D, E**) and preserved proliferative competence within the TME, as reflected by elevated Ki-67 expression (**Figures 5J, K**). Together, these results indicate that the functional advantages conferred by ATG5 OE remain latent when tumor access is limited, but translate into antitumor efficacy once tumor accessibility is improved.

Taken together, these findings identify ATG5-mediated autophagy regulation as an intrinsic mechanism that stabilizes CAR-T cell function under immunosuppressive stress by sustaining inducible autophagy flux and limiting oxidative damage. Rather than functioning as a stand-alone strategy to overcome extrinsic barriers, ATG5-based modulation appears to exert its greatest impact in contexts where tumor access is already partially established. By linking inducible autophagy capacity, redox control, and functional durability in solid tumors, this study refines the conceptual framework for persistence-oriented CAR-T cell engineering.

Despite demonstrating a role for ATG5-mediated autophagy regulation in supporting CAR-T cell functional persistence under solid tumor–associated stress, this study has several limitations. First, cytokine responses were primarily assessed by intracellular cytokine staining, which enables high-resolution single-cell functional analysis but does not capture cumulative cytokine secretion dynamics at the population level. Second, long-term persistence, genomic stability, and safety of ATG5 OE were not evaluated in this study and will require systematic assessment in translational and clinically relevant models. Finally, although the present findings suggest that ATG5-mediated autophagy modulation supports functional persistence under solid tumor–associated stress, its integration with complementary immunomodulatory strategies—such as checkpoint blockade or cytokine signaling modulation—remains to be explored in future studies.

## Data Availability

The raw data supporting the conclusions of this article will be made available by the authors, without undue reservation.
