# Supplementary material for: ATG5-mediated inducible autophagy sustains CAR-T cell durability under solid tumor stress
Source: Front Immunol. 2026 Apr 22;17:1720544. doi: 10.3389/fimmu.2026.1720544 (PMC13143936; doi:10.3389/fimmu.2026.1720544)
Supplement: Supplementary file 1 [file DataSheet1.pdf]

Supplementary Figure 1 (related to figure 1)

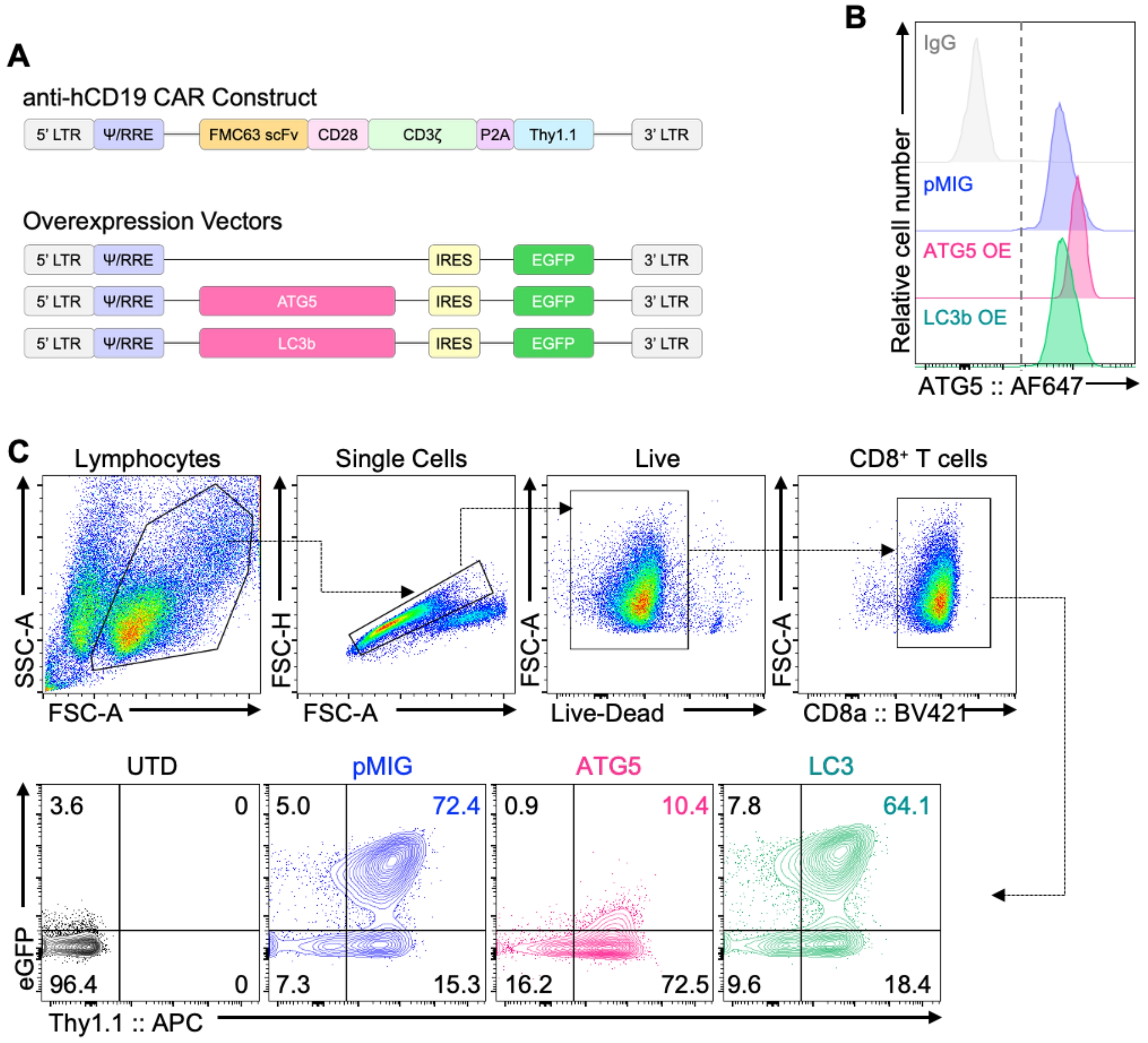

Supplementary Figure 1. Validation of co-expression of CAR and overexpression (OE) construct in mCAR-T cells. (A) Schematic maps of the anti-hCD19 CAR and OE vector constructs (pMIG, ATG5, and LC3b) (B) Representative flow cytometry histograms showing intracellular ATG5 expression in mCAR-T cells transduced with pMIG, ATG5, or LC3b vectors, with IgG used as an isotype control. (C) Gating strategy for identifying Thy1.1$^+$eGFP$^+$ CAR-T cells and representative plots. Representative gating strategy for CAR expression and phenotypic analysis of CD8$^+$ T cells. CAR-T cells were identified as Thy1.1$^+$, and ATG5- or LC3-overexpressing CAR-T cells were distinguished as Thy1.1$^+$eGFP$^+$. All downstream analyses were conducted based on the eGFP$^+$Thy1.1$^+$ population in this study.

Supplementary Figure 2 (related to figure 3)

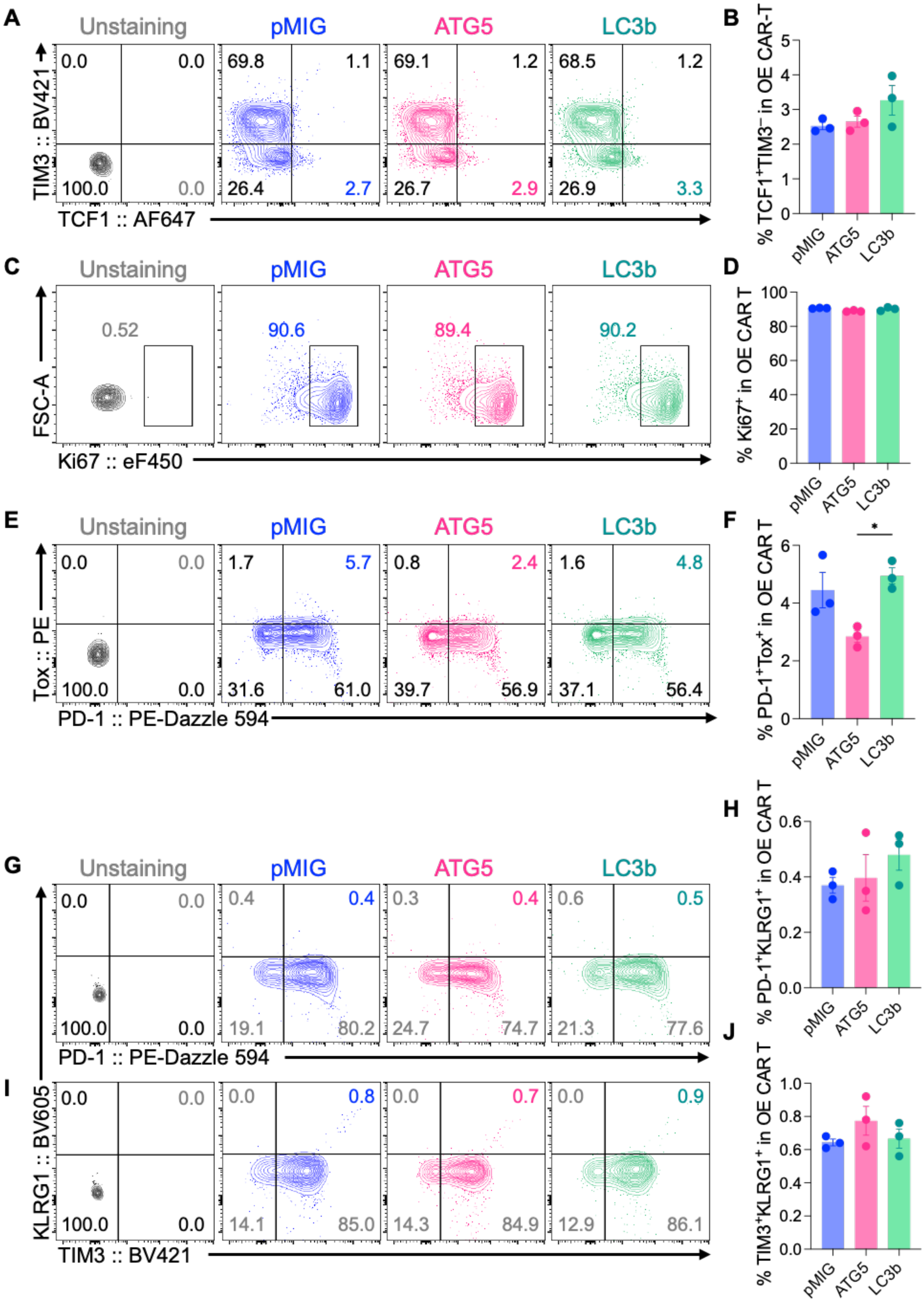

Supplementary Figure 2. Phenotypic profiling of ATG5- and LC3b-overexpressing mCAR-T cells under TGF-β–mediated immunosuppressive conditions. (A, B) TCF1 and TIM-3 expression. (A) Representative flow cytometry plots of unstained controls and mCAR-T cells transduced with pMIG, ATG5, or LC3b constructs. (B) Quantification of the $TCF1^{+}TIM\text{-}3^{-}$ population. (C, D) Proliferative status. (C) Representative flow cytometry plots showing Ki67 expression. (D) Quantification of $Ki67^{+}$ cells. (E, F) Expression of exhaustion-associated markers. (E) Representative flow cytometry plots showing PD-1 and TOX expression. (F) Quantification of $PD\text{-}1^{+}TOX^{+}$ cells. (G–J) Relationship between KLRG1 and inhibitory receptor expression. (G) Representative flow cytometry plots showing PD-1 and KLRG1 expression. (H) Quantification of $PD\text{-}1^{+}KLRG1^{+}$ cells. (I) Representative flow cytometry plots showing TIM-3 and KLRG1 expression. (J) Quantification of $TIM\text{-}3^{+}KLRG1^{+}$ cells. All analyses were performed on $CD8^{+}$ OE mCAR-T cells obtained from the same experimental setting as Figure 3. Individual replicates are shown as dots, and bars indicate mean ± SD. Statistical significance was determined using one-way ANOVA with Tukey's post hoc test. $^{*}p < 0.05$.

Supplementary Figure 3 (related to figure 5)

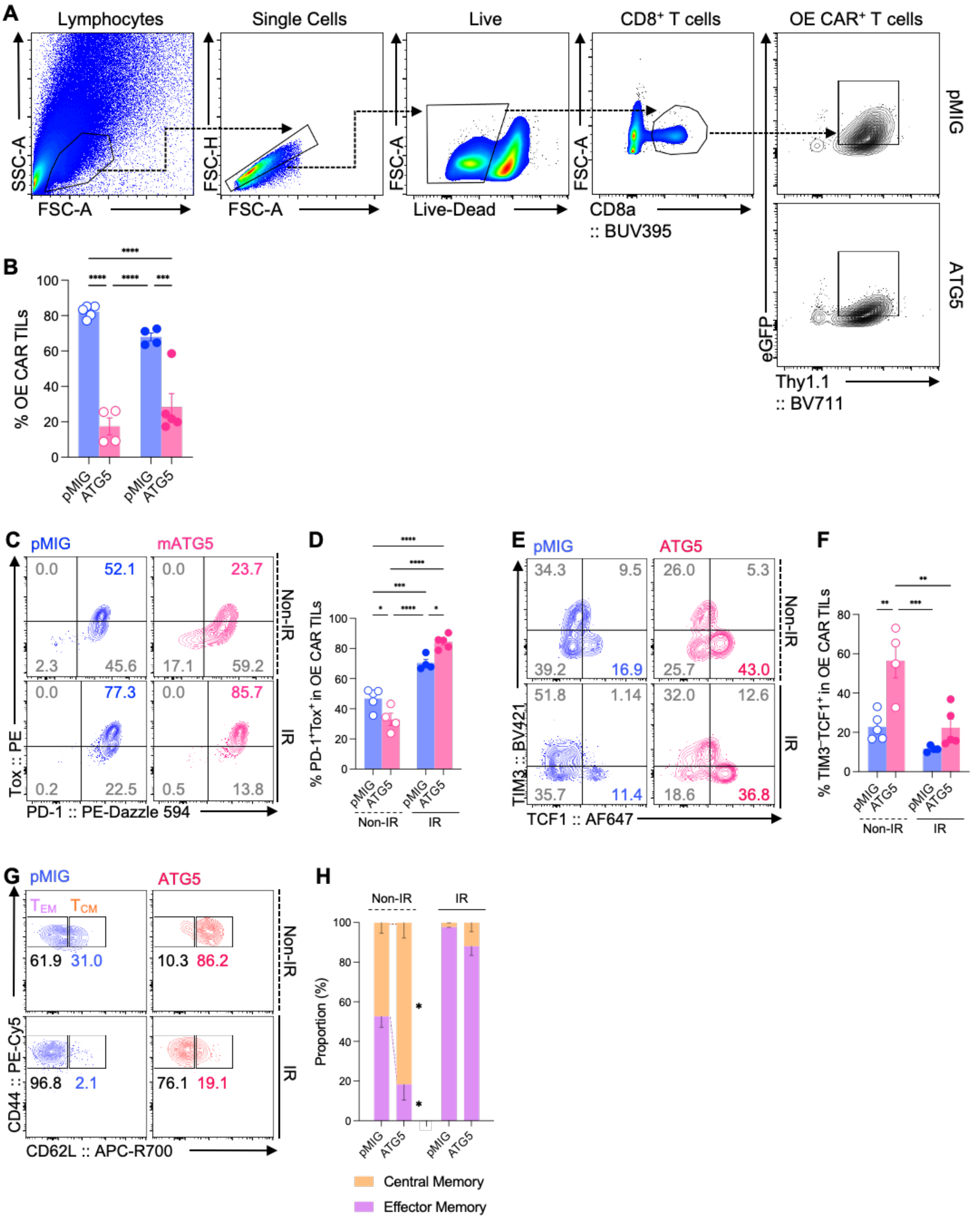

Supplementary Figure 3. Gating strategy and phenotypic characterization of tumor-infiltrating CD8⁺ OE mCAR-T cells under irradiation and non-irradiation conditions. (A) Flow cytometry gating strategy for the identification of CD8⁺ OE mCAR-T cells. Tumor-

infiltrating lymphocytes (TILs) were sequentially gated as lymphocytes (FSC-A vs. SSC-A), single cells (FSC-H vs. FSC-A), live cells (Live/Dead negative), $CD8^+$ T cells ($CD8\alpha^+$), and finally OE CAR-T cells based on Thy1.1 and eGFP expression. Representative gating plots from pMIG and ATG5-OE mCAR-T cells are shown. All TIL analyses in this study were performed using this gating strategy. (B) Frequency of OE CAR-T cells among $CD8^+$ TILs. (C) Representative plots showing the frequency of PD-1^+Tox$^+$ phenotypes. (D) Quantification of PD-1^+Tox$^+$ populations. (E, F) Expression of stem-like T cell markers in $CD8^+$ OE mCAR-TILs. (E) Representative plots showing the frequency of TCF1^+TIM3^- phenotypes. (F) Quantification of TCF1^+TIM3^- populations. Individual replicates are shown as dots, and bars indicate mean ± SEM (n = 4–5). Open dots represent the non-IR group; filled dots represent the IR group. Statistical analysis was performed using an unpaired two-tailed t-test. *$p < 0.05$; **$p < 0.01$; ***$p < 0.001$; ****$p < 0.0001$.
