# Supplementary figures and images for: ATG5-mediated inducible autophagy sustains CAR-T cell durability under solid tumor stress
Source: Front Immunol. 2026 Apr 22;17:1720544. doi: 10.3389/fimmu.2026.1720544 (PMC13143936; doi:10.3389/fimmu.2026.1720544)

Conventional CAR T

ATG5-overexpressing CAR T

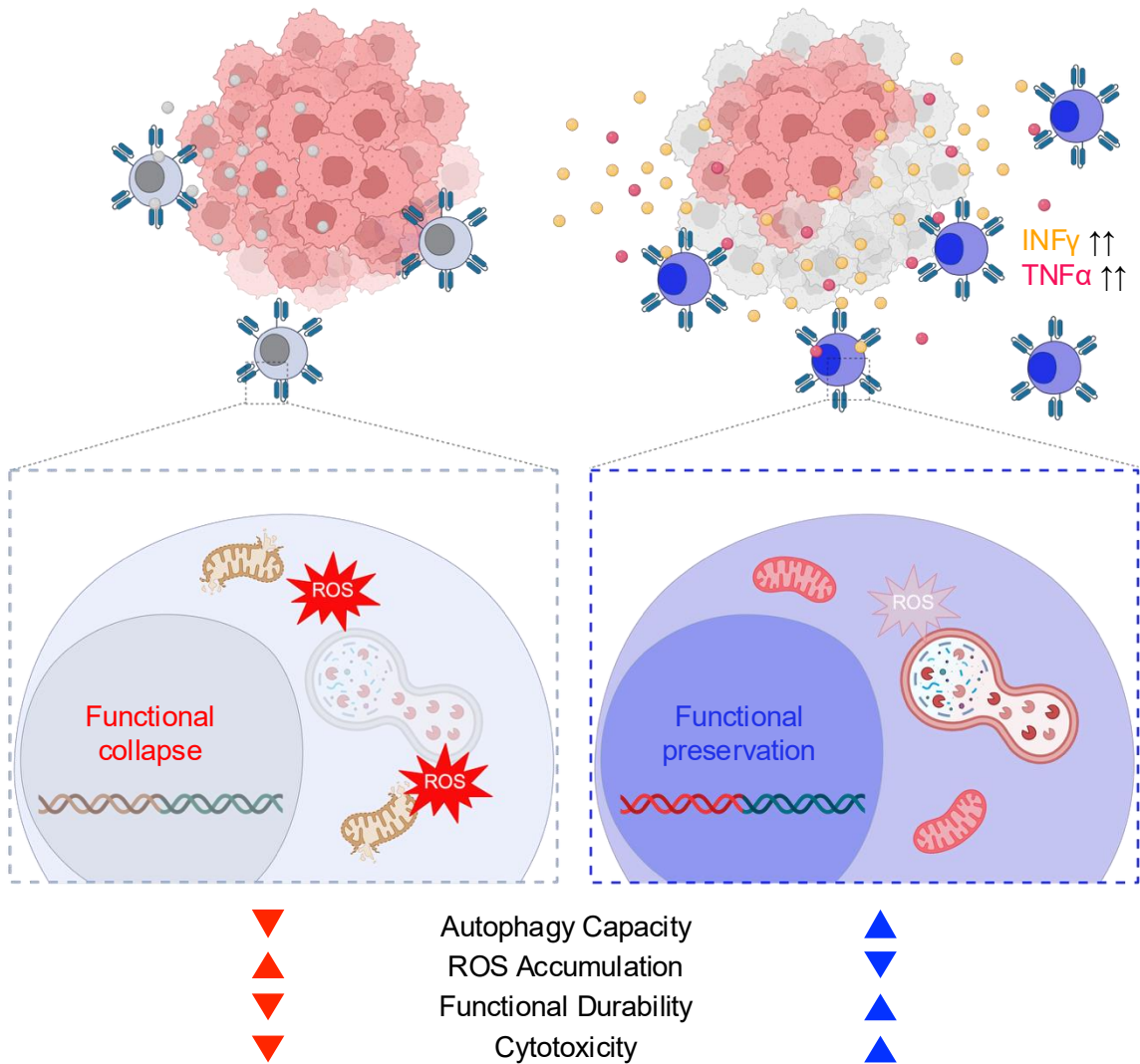

Under tumor-associated stress

Supplement: Supplementary file 2 [file DataSheet2.pdf]
